# Redox Reaction Triggered Nanomotors Based on Soft-Oxometalates With High and Sustained Motility

**DOI:** 10.3389/fchem.2018.00152

**Published:** 2018-05-04

**Authors:** Apabrita Mallick, Abhrajit Laskar, R. Adhikari, Soumyajit Roy

**Affiliations:** ^1^Eco-Friendly Applied Materials Laboratory, College of Chemistry, Central China Normal University, Wuhan, China; ^2^Eco-Friendly Applied Materials Laboratory, Department of Chemistry, Materials Science Centre, Indian Institute of Science Education and Research, Kolkata, India; ^3^The Institute of Mathematical Sciences, Chennai Institute of Technology, Chennai, India; ^4^Department of Applied Mathematics and Theoretical Physics (DAMTP), Centre for Mathematical Sciences, University of Cambridge, Cambridge, United Kingdom

**Keywords:** nanomotors, soft-oxometalates (SOMs), redox process, autonomous motion, chemically powered

## Abstract

The recent interest in self-propulsion raises an immediate challenge in facile and single-step synthesis of active particles. Here, we address this challenge and synthesize soft oxometalate nanomotors that translate ballistically in water using the energy released in a redox reaction of hydrazine fuel with the soft-oxometalates. Our motors reach a maximum speed of 370 body lengths per second and remain motile over a period of approximately 3 days. We report measurements of the speed of a single motor as a function of the concentration of hydrazine. It is also possible to induce a transition from single-particle translation to collective motility with biomimetic bands simply by tuning the loading of the fuel. We rationalize the results from a physicochemical hydrodynamic theory. Our nanomotors may also be used for transport of catalytic materials in harsh chemical environments that would otherwise passivate the active catalyst.

## Introduction

An immediate challenge in the field of soft active matter (Ghose and Adhikari, 2014; Kim et al., 2014; Li et al., 2014a; Sengupta et al., 2014) is to synthesize self-propelling (Ma et al., 2016; Soto et al., 2016; Teo and Pumera, 2016; Teo et al., 2016; Minh et al., 2017; Zhou et al., 2017) in a manner that is facile, preferably single step and that affords a rational control of motility. All such attributes can be built into colloidal charged metal oxide based soft-oxometalates (SOMs) (Roy, 2011, 2014) due to the presence of redox sites on their diffuse interface. Here we trigger SOMs to move by inducing a simple redox reaction on the surface. (Mallick and Roy, 2016, 2018; Mallick et al., 2016).

Autonomously moving micrometer sized particles were first developed in 2002 by Ismagilov et al. (2002) Following the seminal work (Paxton et al., 2004; Kline et al., 2005; Ibele et al., 2009; Duan et al., 2012; Wang et al., 2013) of Sen and Mallouk on catalytic nanomotors (Paxton et al., 2004; Kline et al., 2005; Jurado-Sánchez et al., 2016) many physical mechanisms for rendering particles motile have been explored. These include chemical (Laocharoensuk et al., 2008; Zacharia et al., 2009; Mirkovic et al., 2010; Sattayasamitsathit et al., 2010; Lu et al., 2016), magnetic (Ghosh and Fischer, 2009; Zhang et al., 2010; Gao et al., 2012a; Tottori et al., 2012; Morozov and Leshansky, 2014), electrical (Chang et al., 2008; Calvo-Marzal et al., 2010; Loget and Kuhn, 2010), optical (Eelkema et al., 2006; Roy et al., 2014; Zhang et al., 2017), and ultrasound (Garcia-Gradilla et al., 2013; Esteban-Fernández de Ávila et al., 2016) mechanisms. Of these, the mechanism of chemical propulsion is simplest and most robust but remains limited due to toxicity of the fuel and motility that is limited by lifetime of both the motor and the fuel. Therefore, there is a need to direct the design of chemically powered (Sengupta et al., 2012) nanomotors in a direction that reduces fuel toxicity and increases motility lifetimes. In the present paper, we present a design of a soft-oxometalate (SOM) based nanomotor that is facile, single step, uses relatively non-toxic hydrazine as fuel and has a long motile lifetime of approximately 3 days. Metal oxides like manganese dioxide have been extensively used as nanomotors previously.(Safdar et al., 2015, 2016) To enhance the efficiency of the nanomotors we have synthesized self-assembly of molybdenum oxides which provide increased surface area and multi-metal centres for redox reaction.

The phenomenon of collective motility (Ismagilov et al., 2002) of nanomotors is of considerable significance in the field of nanotechnology as they can perform intended tasks like transport as well as chemical sensing. Sen et al. and Ren et al. reported collective behavior of AgCl (Ibele et al., 2009) and SiO_2_-Pt (Zhang et al., 2015) micromotors respectively in presence of UV light. Our nanomotors also show dynamic schooling behavior in response to high concentration of the fuel which makes them potential devices for transport of catalytic materials.

Chemical propulsion (Paxton et al., 2006; Gao et al., 2012b; Orozco et al., 2013) relies on the conversion of chemical energy to mechanical work performed on the ambient fluid. Typically, chemical reactions on the surface (Ismagilov et al., 2002) induce osmotic flows (Chang et al., 2008; Palacci et al., 2013) in a thin boundary layer surrounding the particle. This osmotic slip flow sets the bulk fluid immediately exterior to the boundary layer into motion. The conservation of mass and momentum, requires a reciprocal motion of the particle in the opposite direction. This chemiosmophoretic mechanism can be realized in a variety of substrates and chemical reactions. The principal requirement for such chemomechanical (Laskar et al., 2013; Sengupta et al., 2013) energy transduction is a reaction that is confined to the motor surface and which produces tangential (Zheng et al., 2013; Brown and Poon, 2014) osmotic stresses that are of sufficient strength and asymmetry to produce macroscopic directional (Patra et al., 2013) fluid flow. The surface of soft-oxometalate (SOM) particles produces osmotic stresses that impart rapid and sustained motility to the particles. Here we show that the redox reaction of hydrazine on the surface of soft-oxometalate (SOM) particles produces osmotic stresses that impart rapid and sustained motility to the particles.

## Materials and methods

### Materials and reagents

The reagents were purchased from commercial sources (Merck) and used without further purification. All glassware was cleaned in an acid bath, base bath and rinsed with isopropanol followed by acetone and kept in an oven for 48 h prior use.

### Synthesis of heptamolybdate {Mo_7_} based soft-oxometalates (SOMs)

Ammonium heptamolybdate tetrahydrate (1,500 mg, 1.213 mmol) was dispersed in HPLC grade water (4 mL) and heated until simmering hot (140°C). A clear dispersion of ammonium heptamolybdate formed, which was then stored in a refrigerator for 10 min. The dispersion was brought to room temperature which scattered light from laser. This aqueous dispersion is at equilibrium between the homogeneous and heterogeneous phases of heptamolybdate. To remove the homogeneous part diethyl ether was added to the dispersion. White precipitate was observed which was separated out by ultra-centrifugation at 3,500 rpm for 15 min. This was washed three times with cold water and ethanol and dried in air. The white solid was re-dispersed in water and was characterized using scanning electron microscopy (SEM), transmission electron microscopy (TEM) and dynamic light scattering (DLS). This dispersion was used for further experiments.

### Preparation of hydrazine sulfate solutions

The solubility of hydrazine sulfate at room temperature was 3.056 mol in 10 mL of water. Calculated amount of hydrazine sulfate was dissolved in 10 mL of distilled water separately to prepare 0.0007, 0.0038, 0.0076, 0.0382, 0.0764, 0.0999, 0.1146, 0.1300, 0.1375, 0.1528, 0.1910, 0.2292, 0.2674, and 0.3056 mol L^−1^ hydrazine sulfate solutions.

### Characterization using dynamic light scattering (DLS) measurement

0.5 mL of heptamolybdate SOM dispersion was diluted with 10 mL of deionised water. This dispersion was irradiated with a hand held laser pointer of wavelength 635 nm which resulted in the scattering of a single well-defined line. The resulting dispersion was then subject to DLS experiment using Malvern Zetasizer. This was repeated for all dispersions of SOM and SiO_2_ with addition of hydrazine as well.

### Characterization using transmission electron microscopy (TEM)

**Figure 1:**
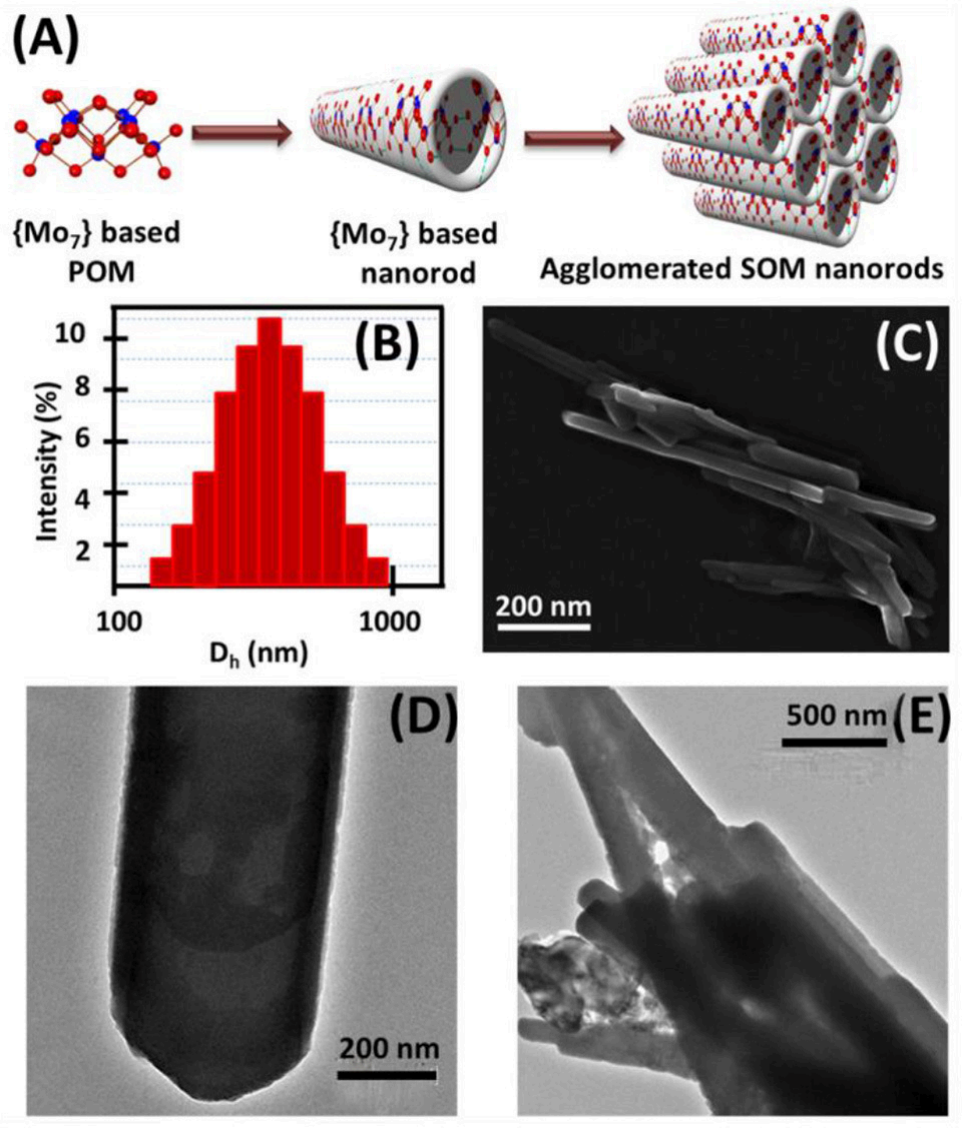
**(A)** Schematic of the formation of SOM nanorods and their aggregates, **(B)** DLS study of SOM dispersion, **(C)** SEM image of aggregated SOM nanorods and TEM images of **(D)** single SOM nanorod, **(E)** aggregated SOM nanorods.

The characterization of isolated SOM and collective SOMs were primarily done by the use of a Tecnai 20 transmission electron microscope (FEI Company) operated at an accelerating voltage of 200 kV. The TEM micrographs were processed using SIS software (Soft Imaging System). The TEM images are illustrated in Figure 1.

### Characterization using scanning electron microscopy (SEM)

The characterization of SOMs was done using SUPRA 55 VP-41-32 instrument with the SmartSEM version 5.05 Zeiss software. The SEM image is shown in Figure 1.

### Study of surface kinetics by electron absorption spectrophotometry (EAS)

EAS spectra were recorded using Shimadzu UV spectrophotometer (UV 1,800). 100 μL of hydrazine sulfate solution of known concentration was added to 100 μL of ammonium heptamolybdate SOM dispersion in a UV cuvette, diluted with distilled water to 3 mL, the kinetics mode was activated in UVProbe 2.5 and the kinetics study was carried out directly in the spectrophotometer for a time period of 5 h. The kinetics study was repeated for all the concentrations of hydrazine sulfate solution following the same procedure.

### Microscopy using inverted fluorescence microscope

An Olympus IX81epi fluorescence microscope with a motorized stage was used for recording videos. 75 × 25 × 1.45 mm micro concavity slides with polished spherical concavities of 15 mm diameter × 0.5 mm depth and a 22 × 40 mm cover glass were cleaned with methanol and dried to remove any unwanted adsorbed material. The SOM dispersion (100 μL) was placed in the cavity of the slide. 100 μL of hydrazine (known concentration, freshly prepared) was added to the SOM dispersion using a 100 μL micropipette. The cavity was then sealed with the cover slip in order to prevent any air convection current. The whole set-up was then placed on the microscope scanning stage and the stage was controlled using a joystick. The dispersion which was initially colorless gradually turned blue after addition of hydrazine sulfate solution. The microscope was focused at 40X objective and the videos were recorded using DSIC camera at a rate of 10 frames per second.

### Analysis using ImageJ and TrackPy

**Figure 2:**
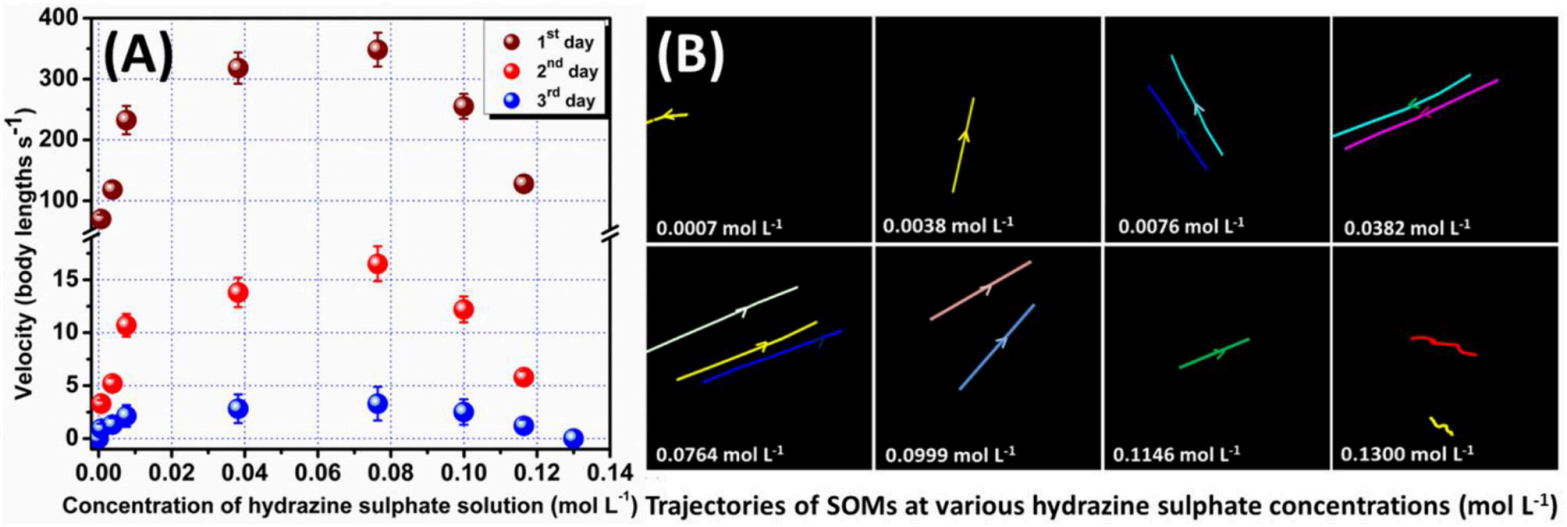
**(A)** Plot of velocity of SOMs at different hydrazine concentrations on 3 days respectively with error bar. **(B)** Trajectories of the moving SOMs indicated by the colored lines at different hydrazine concentrations for a time period of 0.5 s on 1st day. Arrows denote direction of motion.

The raw image sequence in. TIFF format was converted to. AVI form using ImageJ. ImageJ (Rasband, 2011; Schneider et al., 2012) is a Java based image processing and analysis software (original name, NIH image) which is available in public domain and allows analysis of multiple images sharing a single window (video). From these videos each frame was separately taken and the SOM particles in that frame were analyzed manually. That is, the co-ordinates of position of a SOM particle was measured over three frames, the body length of that particular SOM was taken into account (as SOMs are of varying size), time gap between the frames was noted and using these velocity of each SOM was calculated. This procedure was repeated for a number of SOMs in each video corresponding to a particular hydrazine concentration. The velocities were found to be coherent. However, the average of these velocities was considered as the velocity of the SOM at that particular hydrazine concentration. The same procedure was carried out for all hydrazine concentrations. The velocities of SOM were plotted against concentration of hydrazine (Figure 2).

Trackpy is a python packaging tool used for particle tracking. Using this code each video was analyzed and trajectories of SOM were obtained. Trackpy first identified SOM particles in each frame with selective filters in it and then connected the frames in which a SOM particle was present to obtain the particle trajectories which were found to be similar to that obtained with ImageJ (Figure 2, given later).

## Results and discussion

### Surface reaction chemistry

We now describe the details of the surface reaction chemistry we use to obtain particle motility. We disperse ammonium heptamolybdate tetrahydrate in water and heat it for few min till boiling followed by cooling. Linear {Mo_7_} building blocks self-assemble due to charge-regulation (Verhoeff et al., 2007), counter-ion mediated attraction as well as water-bridged hydrogen bonding (Yin et al., 2012) in aqueous solution and form soft-oxometalate nanorods which are shown schematically in Figure 1A. SEM and TEM images reveal that thin and well-defined SOM nanorods of {Mo_7_} (Figures 1C,D and Supplementary Figure 1) are formed. The size of the nanorods is approximately 200 ± 80 nm from SEM and TEM images which is in consistence with the hydrodynamic diameter (approximately 500 nm) from the DLS experiments (Figure 1B). The increase in particle size may be due to surrounding solvent molecules or agglomeration amongst themselves. In aqueous solution counter-ions directly bind covalently to these anionic rods which results in inter-rod attractions (Ryu et al., 2008) and these tubes coalesce to form divergent bundles with a conical apex as revealed from SEM and TEM images in Figures 1C,E respectively.

These particles of soft-oxometalate can be rendered motile through a redox reaction on their surface fuelled by aqueous solution of hydrazine sulfate (N_2_H_6_SO_4_). In heptamolybdate SOMs the oxidation state of Mo center is +VI. When hydrazine is added to the dispersion of SOMs, it gets oxidized to nitrogen (N_2_) which has been confirmed from GC-MS (Supplementary Figure 6) and some Mo (VI) centers of SOM readily get reduced to Mo (V) through the following reaction (Muller et al., 2004):

(1) $$\begin{array}{l} {\text{Mo}}_{7}^{\text{VI}}{\text{O}}_{24}^{6-}+{\text{N}}_{2}{\text{H}}_{6}^{2+}+{\text{H}}^{+}\to \left\{{\text{Mo}}^{\text{VI}}{\text{Mo}}^{\text{V}}\right\}\left(\text{brown}\right)\\ +{\text{N}}_{2}\left(\text{g}\right)+{\text{H}}_{2}\text{O}. \end{array}$$

Owing to micro-environmental effect, the acidity at the vicinity of the {Mo_7_} SOMs is higher than the bulk fluid. Thus, these SOMs are inherently greener which leads us to avoid addition of any mineral acids externally, though we still get the efficiency of acidic pH. Brown coloration has been observed in the reaction medium after due to Mo (V) Mo (VI) inter valence charge transfer (IVCT) which has been analyzed with EAS later. The enhanced concentration of nitrogen near the SOM surface produces a gradient of chemical potential which must lead to an osmotic slip flow tangential to the SOM surface. The standard chemiosmophoretic mechanism, then, propels the particle in a direction opposite to the osmotic slip flow. The completion of the above reaction from SOM nanotubes to brown amorphous powder of Mo takes approximately 4–5 days, thereby rendering the motor with a motile life of at least 3 days though with much less velocity (Figure 2A and Supplementary Table 1). The brown powder has been characterized with EAS and FT-IR (Supplementary Figure 3). They are found to be similar but the absence of acetate cannot be confirmed from these spectra because the band at 1,400 cm^−1^ arises from δ_as_(${\text{NH}}_{4}^{+}$) and according to literature the bands from acetate (δ_CH3_ and γ_coo_) if present, will be masked. From EAS (Supplementary Figure 3A), a band is observed at 450 nm which implies the brown color formed due to intervalence charge transfer (IVCT) between Mo(V) and Mo(VI) centers. Cyclic voltammetry experiments (Supplementary Figure 4) show a reduction peak at −0.57 V and an oxidation peak at −0.4 V due to Mo(V)/Mo(VI) (Clemente-León et al., 2007). Further to count the number of Mo(V) centers in the product we have performed cerimetric titrations the results of which have been provided in details in the Supporting Information (Supplementary Figure 5). The results show 8.4 mL of Ce(IV) is required to oxidize the Mo(V) centers to Mo(VI) which corresponds to 2.52 × 10^19^ electrons. So in 45 mg of the brown product 2.52 × 10^19^ Mo(V) centers are present.

### Control of motility

**Figure 3:**
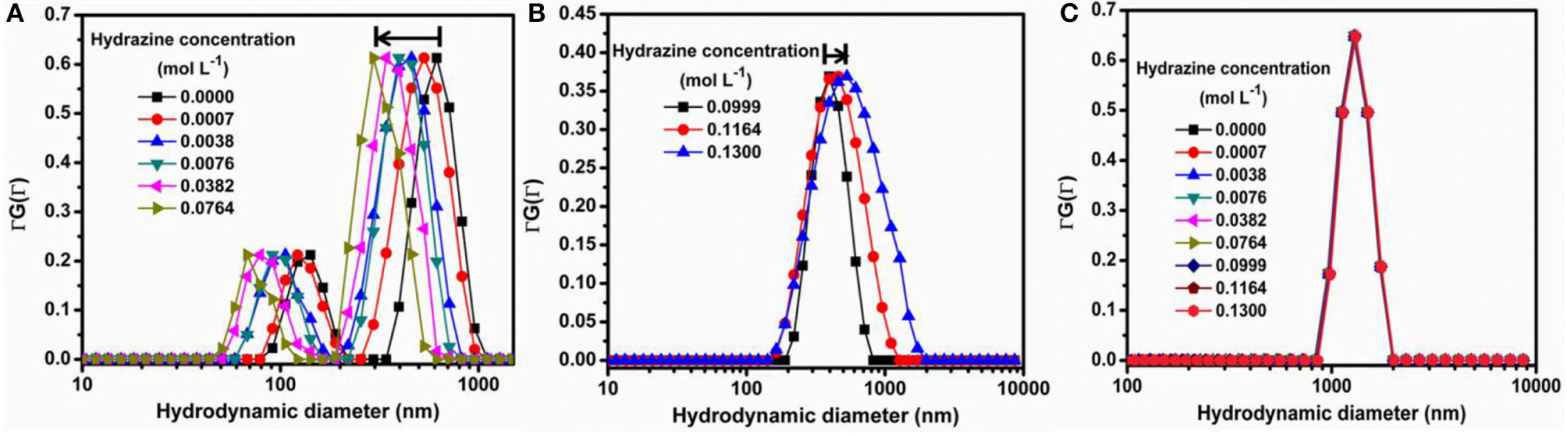
Dynamic Light Scattering (DLS) experiments for **(A)** SOMs in 0.0000–0.0764 mol L^−1^ hydrazine showing left shift, **(B)** SOMs in 0.0999–0.1300 mol L^−1^ hydrazine showing right shift, **(C)** SiO_2_ in 0.0000–0.1300 mol L^−1^ hydrazine showing negligible shift.

The possibility of air convection current affecting the motion of the nanomotors has been eliminated by performing the microscopy in a sealed chamber which has been described in the experimental section. Also, to confirm that the motion of SOMs is not an artifact of fluid convection current we have performed control experiments replacing SOMs with silica particles without altering other experimental conditions (Supplementary Videos). Silica particles do not show any directional motion unlike the SOM particles. This is also supported from the dynamic light scattering (DLS) experiments where a clear shift of hydrodynamic diameter is seen for SOMs in presence of hydrazine but for SiO_2_ particles the hydrodynamic diameter remains almost same in different concentrations of hydrazine (Figure 3). The speed of isolated nanomotors can be tuned through the loading of hydrazine. We compute the nanomotor speed from particle tracking of trajectories using TrackPy (Allan et al., 2014). Our nanomotors reach a maximum speed of (348 ± 22) body lengths per second as the hydrazine concentration reaches approximately 0.0764 mol L^−1^, as shown in Figure 2. In comparison, Au-Pt nanorods (Paxton et al., 2004) move at 10 body lengths per second, catalytic Ir/SiO_2_ micromotors (Gao et al., 2014) move at 20 body lengths per second, hydrogen bubble propelled PANI/Zn microrockets (Gao et al., 2011) move at 100 body lengths per second while PEDOT/Pt microbots (Gao et al., 2012c) move at 480 body lengths per second. Our motors, therefore, are amongst the fastest (normalized by their size) of currently reported active particles.

In order to quantify the effect of loading of chemical fuel on motion of active SOMs we now increase in steps, the concentration of our fuel, hydrazine. With increase in hydrazine sulfate concentration up to 0.0746 mol L^−1^ the velocity of SOMs also increase exponentially as is evident from Figure 2. At 0.0764 mol L^−1^ of hydrazine sulfate concentration, velocity reaches a value of 348 ± 22 body lengths s^−1^ (Supplementary video). Past that concentration, the velocity of SOM decreases even with increase in hydrazine concentration and at 0.13 mol L^−1^ of hydrazine sulfate no significant motion of SOM is observed. These data are in well agreement with the diffusion coefficient and DLS distribution curves where the first a left shift is observed followed by a right shift indicating initial increase in velocity of SOMs with increasing concentration of fuel followed by the decrease in their velocity (Figure 3 and Supplementary Figure 2).

The nonmonoticity of the propulsion velocity as a function of hydrazine concentration has to be explained in terms of the changing nature of osmotic stress at the particle surface. The SOM particle has considerable asymmetry in terms of its surface structure. The anterior end of the SOM is capped like a pencil while the posterior end consists of a series of rod like protrusions. We believe that these exposed ends of the rods provide a more accessible reaction surface than the capped end. Therefore, hydrazine preferentially reacts at the posterior open end. At low concentrations, the spatial asymmetry of the intensity of chemical reaction produces a corresponding asymmetry in the distribution of osmotic stress. This leads to particle propulsion. With increasing hydrazine concentration, the amount of reaction increases more rapidly than the spatial asymmetry decreases. This leads to an overall increase in propulsion speed. However, at a critical concentration, the asymmetry decreases more rapidly than the increase in reaction centers. Thus, although more fuel is available, the reaction takes place across the entire body of the SOMs and this leads to a symmetric distribution of the osmotic stress. The particle can no longer propel in the resulting symmetrically distributed flow, but, it does nonetheless produce a fluid flow around itself. On general grounds the flow must be as seen in dipoles and quadrupoles as seen in **Figure 5**. Any micro-swimmer that swims cannot have any net external force or net external torque. The swimming motion and generation of fluid flow associated with it, at low Reynolds number environment can be understood through an additional slip velocity on the surface of the micro-swimmer (Singh et al., 2015). Exploiting the linearity of Stokes flow (Navier-Stokes Equation in the viscous limit) and boundary conditions without isolating the details of internal mechanism, it has been shown that any generic slip velocity can be decomposed into irreducible multipoles (Singh et al., 2015). Different irreducible multipoles represent different modes of stress-generation and associate fluid-flows. These fluid-flows contain different internal structures and decay algebraically as the distance from the source decreases. The order of the algebraic decay increases with the increase of order of multipole. Using similar approach, the flow produced by motile neutrally-buoyant SOM must be seen as a combination of Stokes-dipole or stresslet and potential-dipole or degenerate quadrupole at leading order. We note that stresslet does not contribute in self-propulsion and the associate flowfield decays as 1/r^2^, whereas, degenerate quadrupole produces leading contribution for self-propulsion and the associated flow field decays as 1/r^3^, where r is the distance of field point from the source point.

**Figure 4:**
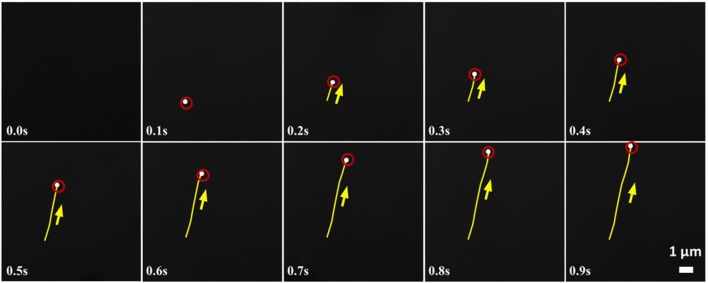
Time lapse images of a moving heptamolybdate SOM in 0.0038 mol L^−1^ of hydrazine sulfate. Direction of motion is shown by the arrows and yellow lines indicate the trace lines i.e., the position of SOM from the initial position at 0 s. Red circles help locating the white SOMs.

**Figure 5:**
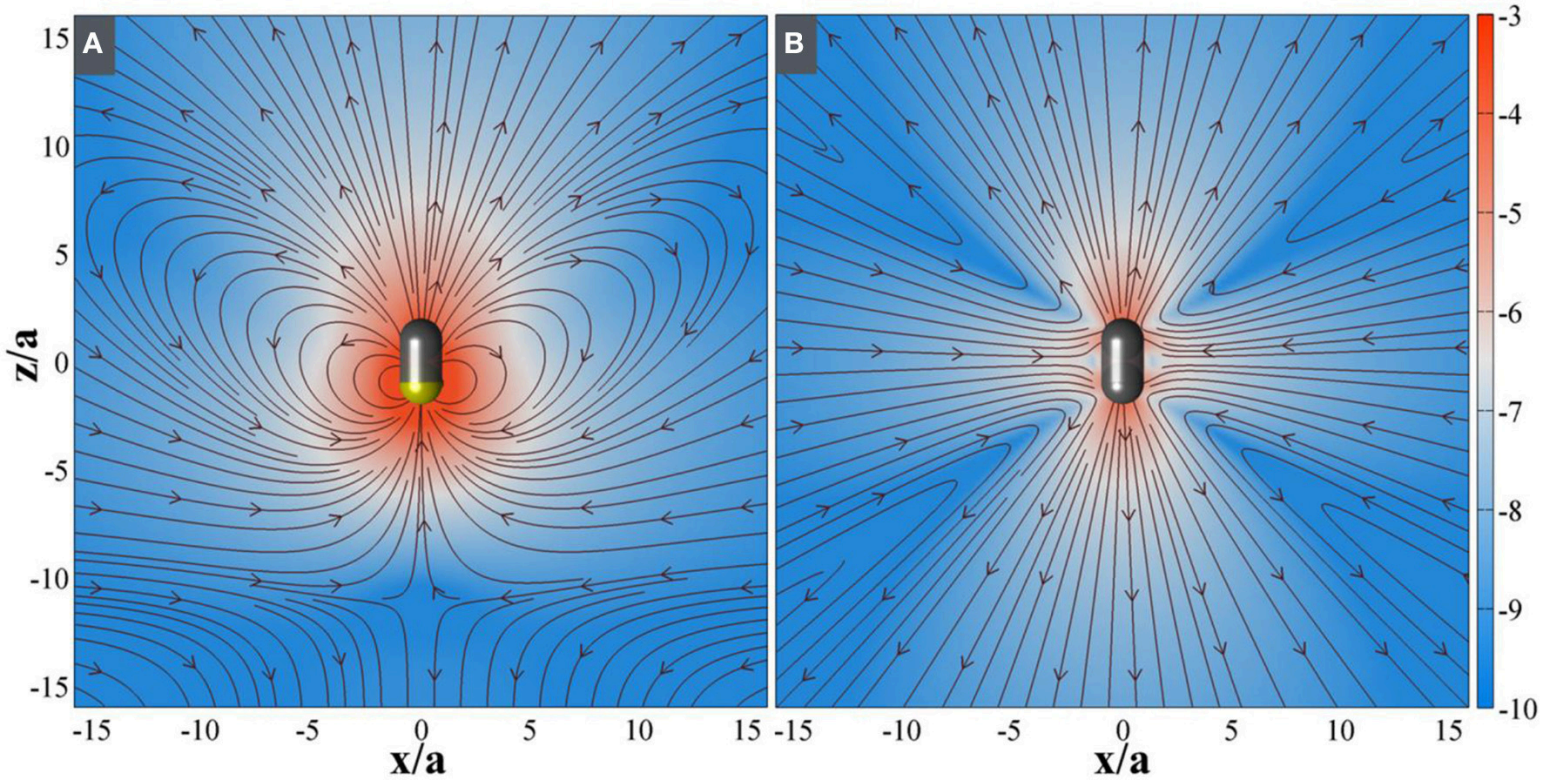
Computed fluid flow around active SOM when **(A)** propulsive and **(B)** stalled. The streamlines show the direction of fluid motion and the background is pseudo-colored with logarithm of magnitude of the fluid velocity. In the **(A)**, the flow around a SOM breaks the symmetry due to asymmetric surface slip and thus induces a propulsion motion. In the **(B)** the flow around a SOM restores the symmetry due to reduction of asymmetric surface slip both at very low, and high hydrazine concentration which results in decreasing propulsion efficiency.

In our experiment, we observe a transition from a symmetry-broken motile state of activity to a symmetric stalled state of activity as the reaction increases with the increase of hydrazine. Below the critical concentration of Hydrazine, we have observed that the SOMs are moving very fast in a particular direction (Figure 4). Therefore, keeping the strength of the stresslet approximately 10% (it can be even smaller), of the potential-dipole, the flow-field around the SOM can be predicted, as seen in Figure 5. Above the critical concentration of hydrazine, the spatial asymmetry of distribution of product and correspondingly distribution of osmotic stress decreases which in turn, leads to suppression of motile potential-dipole mode. Therefore, stalled SOM at high concentration of Hydrazine can only have stresslet at leading order and the associated flow-field is displayed in Figure 5.

### Surface tension and the direction of propulsion of the SOM nanomotors

**Figure 6:**
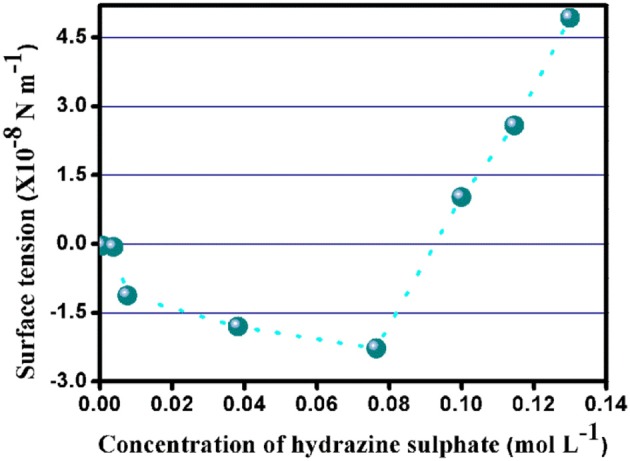
Plot of surface tension at varying hydrazine sulfate concentrations.

The surface tension guides the direction of propulsion of the nanomotors i.e.,; it determines whether the motors tend to move along the gradient of the fuel or the gas evolved due to the interactions between the SOM surface and the fuel. From the calculation of surface tension according to Kelvin's equation (from Figure 6 and Supplementary Table 3) it can be seen that the surface tension has negative values upto 0.0764 mol L^−1^ of hydrazine sulfate concentration.

Beyond this concentration, the surface tension becomes positive. From the negative values of surface tension we may infer that the SOM nanomotors move along the direction of the gas evolved. With the increase in hydrazine concentration the value of surface tension becomes positive which leads to the movement of the motors in the opposite direction i.e.,; along the direction of the concentration gradient of the fuel.

### Collective motion

SOMs are charged particles with huge number of negative charges residing on the surface. On addition of hydrazine to the {Mo_7_} SOMs, some Mo(VI) centers are reduced to Mo(V) (from equation 1) which generates more negative charge on the surface, enhancing their chance of agglomeration. SOMs are highly asymmetric rods (surface area at the anterior end is lesser than the posterior part as shown in Figure 1E), resulting in energy difference between the surfaces. To minimize the overall charge the SOM particles agglomerate to form bundles of such rods. This is more prominent at higher concentrations of hydrazine which stems schooling behavior in SOMs.

**Figure 7:**
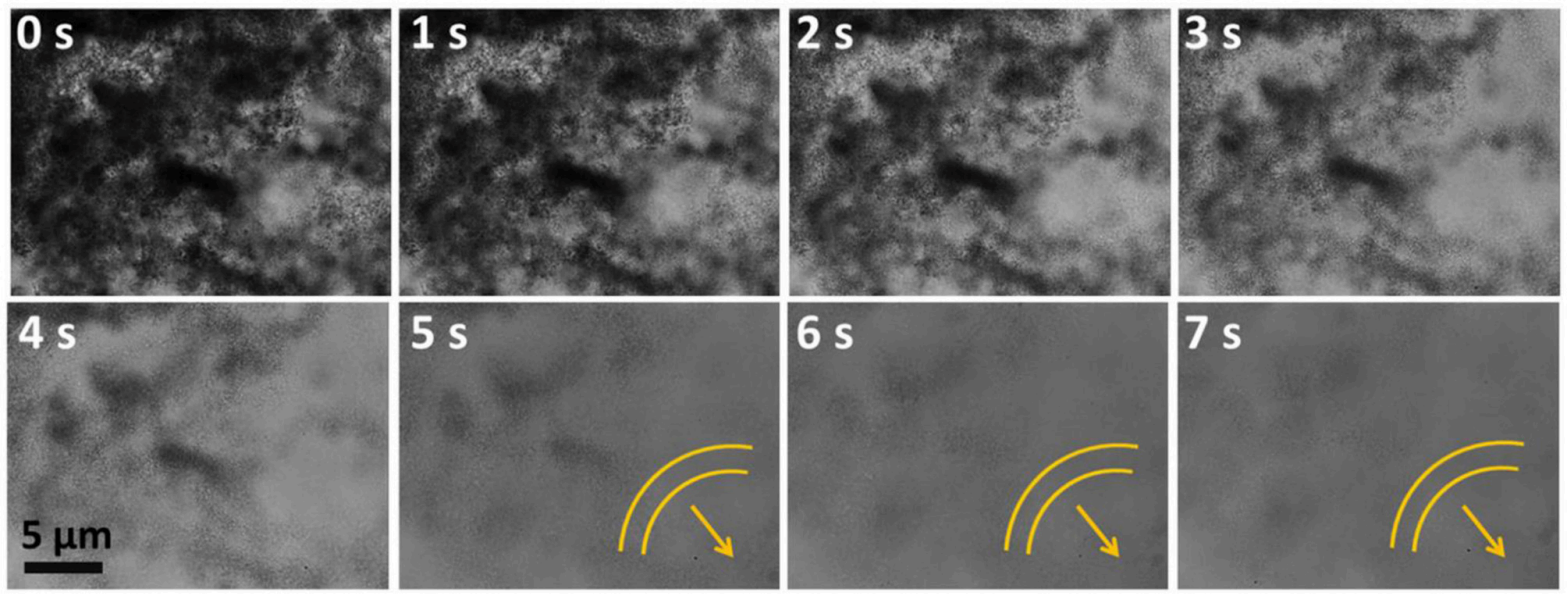
Snapshots of collective motion of SOMs at 0.1910 mol L^−1^ concentration of hydrazine sulfate from 0 to 7 s. Arrows denote the band formation.

We now discuss the transition to collective motion that takes place when the fluid flow around the particles becomes symmetric from hydrodynamic viewpoint. When individual particle motility ceases due the symmetric distribution of osmotic stresses, there is still, as we discussed, hydrodynamic flow around the particle. On general grounds, it can be argued that this hydrodynamic flow must have dipolar symmetry. Particles aligned along the extensional axis of the flow are convected away from each other, while particles aligned along the contractile axis are convected toward each other. For sufficient strength of flow, this can lead to a flow-induced clumping of particles. The distribution of osmotic stresses in such a collection of particles, viewed as an aggregate entity, need no longer be symmetric. The aggregate as a whole can then propel itself, as seen from Figure 7, though at a much reduced speed. We see a clear signature of this transition to collective motility as a function of hydrazine volume fraction. Particles aggregate and travel in collective bands at a fraction of the speed of the individual particles. We believe such motion can be harnessed for transport of catalytic materials to large spatially distributed regions.

### Surface reaction kinetics

**Figure 8:**
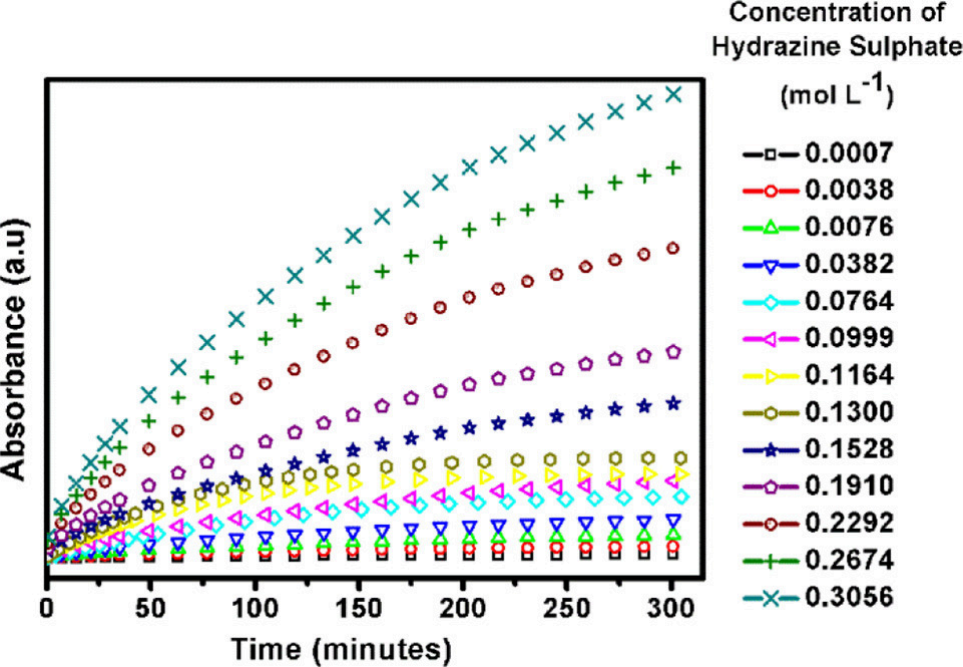
Reaction kinetics of SOM-hydrazine sulfate system obtained from electron absorption spectroscopy (EAS) studies.

In an effort to understand the surface reaction kinetics on the soft-oxometalate surface we have performed the electron absorption spectroscopy (EAS) studies. Loading of different concentrations of fuel, i.e.,; hydrazine sulfate leads to similar kind of kinetics with an initial increase in absorbance and then saturation. The plot of reaction rate vs. reactant concentration reveals how much reactant gets adsorbed on the soft- oxometalate surface, and from Figure 8 we find that the kinetics resembles Michaeles-Menten kinetics which is one of the well-known enzyme kinetics. From the graph we may imply that the number of sites on SOM surface adsorbed by the gas increases initially but after a critical value, most of the sites are occupied and even though the amount of reactant increases the fraction of sites occupied by gas remains almost constant.

### Power conversion efficiency of the SOM nanomotors

The power conversion efficiency (η) (Wang et al., 2013) of these nanomotors can be defined as:

$$\begin{array}{l} \eta =\;\frac{P_{mech}}{P_{chem}} \end{array}$$

where, ***P*_*mech*_** is the mechanical energy output and is determined by the equation:

$$\begin{array}{l} P_{mech}\;=\;F_{drag}v=fv^{2}=\;\gamma v^{2} \end{array}$$

and ***P*_*chem*_** is the chemical energy input provided by the redox reaction and is found out from the equation:

$$\begin{array}{l} P_{chem}=n\Delta_{r}G_{\gamma } \end{array}$$

For cylinders,

$$\begin{array}{l} \gamma =\frac{2\pi \mu L}{\text{ln}\left(\frac{L}{R}\right)-0.72} \end{array}$$

**Figure 9:**
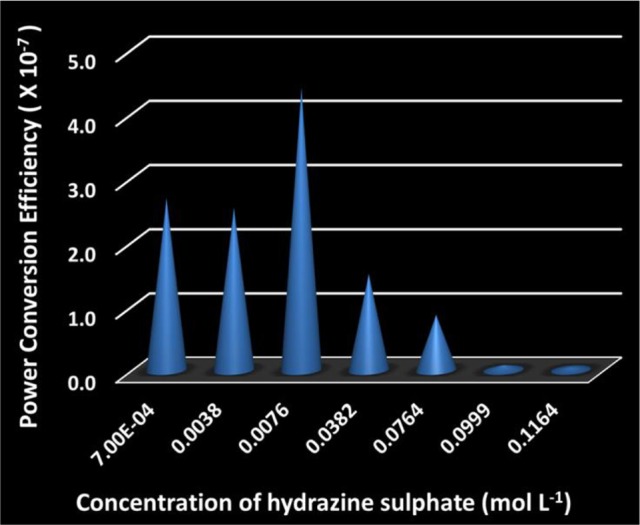
Plot of power efficiency of SOMs at different hydrazine sulfate concentrations.

Here, ***F***_***drag***_ is the drag force on the cylindrical SOM, γ is the drag coefficient, μ is the dynamic viscosity of water, ***L*** is the length of SOM, ***R*** is its radius, ***v*** is the motor speed, ***n*** is the nitrogen gas evolution rate in units of mol/(SOM·s) and **Δ**_*r*_***G***_**γ**_ is the Gibbs free energy of the decomposition of dithionite. We calculate the values of ***P***_***mech***_ and ***P***_***chem***_ and find the energy efficiency of SOM nanomotors which is demonstrated in Figure 9. The power conversion efficiencies of the SOM nanomotors are found to be in the order of 10^−7^ (Supplementary Table 2) which is sufficiently high in the field of nanomotors (Wang et al., 2013). The highest efficiency is obtained for the hydrazine concentration of 0.0076 mol L^−1^ which comes around 4.12 × 10^−7^.

## Conclusion

To summarize, in our current work we have used molybdenum based soft-oxometalates (SOMs) and employed hydrazine sulfate fuel to induce motility in these SOM nanorods. The directional motility in these SOMs stems from the evolution of the nitrogen gas due to the reaction between {Mo_7_} surface and the fuel where hydrazine is oxidized to nitrogen gas and heptamolybdate type SOMs are reduced to molybdenum blue SOMs. Due to this reaction an osmotic boundary is generated between product i.e.,; the gas and the unreacted SOM surface. The chemical potential gradient between the surface of the heptamolybdate SOMs and the osmotic interface gives rise to a slip velocity to the SOMs propelling them with a high propulsion velocity of 369 body lengths per second. The velocity of the SOMs again decreases due to the viscous drag induced by nitrogen on the SOM surface. The velocity finally drops to to zero when the viscous drag annuls the slip velocity. Observation of bands in our system which is totally non-living has been explained here. Such facile synthesis and high propulsion velocity of heptamolybdate SOMs hold considerable importance in the field of active matter and can be used in future for transport (del Mercato et al., 2014; Manna et al., 2017) and catalysis (Li et al., 2014b) after properly controlling their direction of motion. We can further use these active SOMs for preparing nanocarpets utilizing their high velocity by immobilizing heptamolybdate on graphene sheets. Also we can use them for preparing raft by proper alignment of the SOM nanorods.

## Author contributions

SR conceived, designed the experiments with RA. AM performed the experiments and characterizations. AM, AL, RA, and SR analyzed the data. AL and RA provided the hydrodymanic model. SR and RA wrote the manuscript with inputs from AM and AL. All authors read and approved the paper.

### Conflict of interest statement

The authors declare that the research was conducted in the absence of any commercial or financial relationships that could be construed as a potential conflict of interest.
