# Supplementary material for: Redox Reaction Triggered Nanomotors Based on Soft-Oxometalates With High and Sustained Motility
Source: Front Chem. 2018 May 4;6:152. doi: 10.3389/fchem.2018.00152 (PMC5946003; doi:10.3389/fchem.2018.00152)
Supplement: Supplementary file 16 [file Data_Sheet_1.pdf]

Supplementary Material

Redox reaction triggered nanomotors based on soft-oxometalates with high and sustained motility

Apabrita Mallick, Abhrajit Laskar, R. Adhikari*, Soumyajit Roy*

*** Correspondence:** Dr. Soumyajit Roy: s.roy@iiserkol.ac.in, Dr. R. Adhikari, rjoy@imsc.res.in

(a) Cryo-TEM of heptamolybdate SOM rod

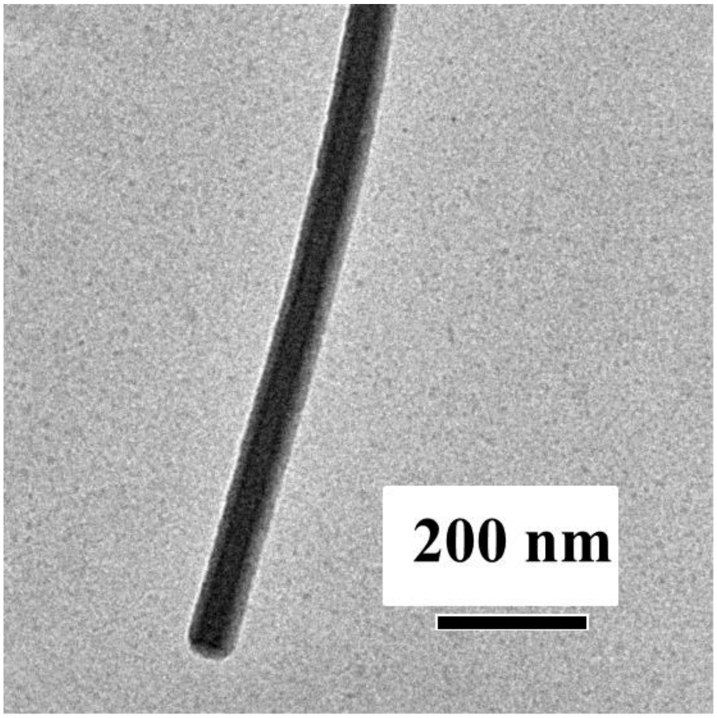

Supplementary Figure 1: Cryo-TEM image of heptamolybdate SOM rod

(b) Velocities of the heptamolybdate SOM nanomotors at different concentrations of hydrazine sulphate solutions:

Applying temperature correction, the solubility of hydrazine sulphate was found to be 3.056 mol per 10 mL of distilled water at room temperature. So we have used this solution of hydrazine sulphate as the maximum concentration of fuel in our experiments. To understand the effect of fuel on the velocity of the SOM nanomotors we have used varying concentrations of hydrazine sulphate which is shown graphically in the main paper. The exact velocity values with the error are tabulated here.

Supplementary Table 1: Velocities of the heptamolybdate SOM nanomotors at different concentrations of hydrazine sulphate solutions:

**Concentration of hydrazine sulphate (mol L^{-1})**	**Velocity of SOM nanomotors on day 1 (bodylengths s^{-1})**	**Velocity of SOM nanomotors on day 2 (bodylengths s^{-1})**	**Velocity of SOM nanomotors on day 3 (bodylengths s^{-1})**
0.0007	69.76 ± 6.97	3.3 ± 0.13	0.94 ± 0.16
0.0038	118.25 ± 9.82	5.2 ± 0.32	1.32 ± 0.35
0.0076	232.40 ± 17.24	10.7 ± 0.52	2.14 ± 0.62
0.0382	317.92 ± 20.27	13.8 ± 1.08	2.82 ± 1.21
0.0764	348.23 ± 22.16	16.5 ± 1.37	3.3 ± 1.50
0.0999	255.16 ± 18.32	12.2 ± 0.98	2.51 ± 1.02
0.1164	127.65 ± 11.21	5.8 ± 0.41	1.21 ± 0.32
0.1300	0	0	0

At the concentration of 0.1300 mol L^{-1} hydrazine sulphate the motion stalls. Beyond the concentration of 0.1300 mol L^{-1} we observe collective motion of the SOMs. So we have not included those concentrations (0.1375-0.3056 mol L^{-1}) in the above table.

(c) Diffusion coefficient of SOMs at different concentrations of hydrazine:

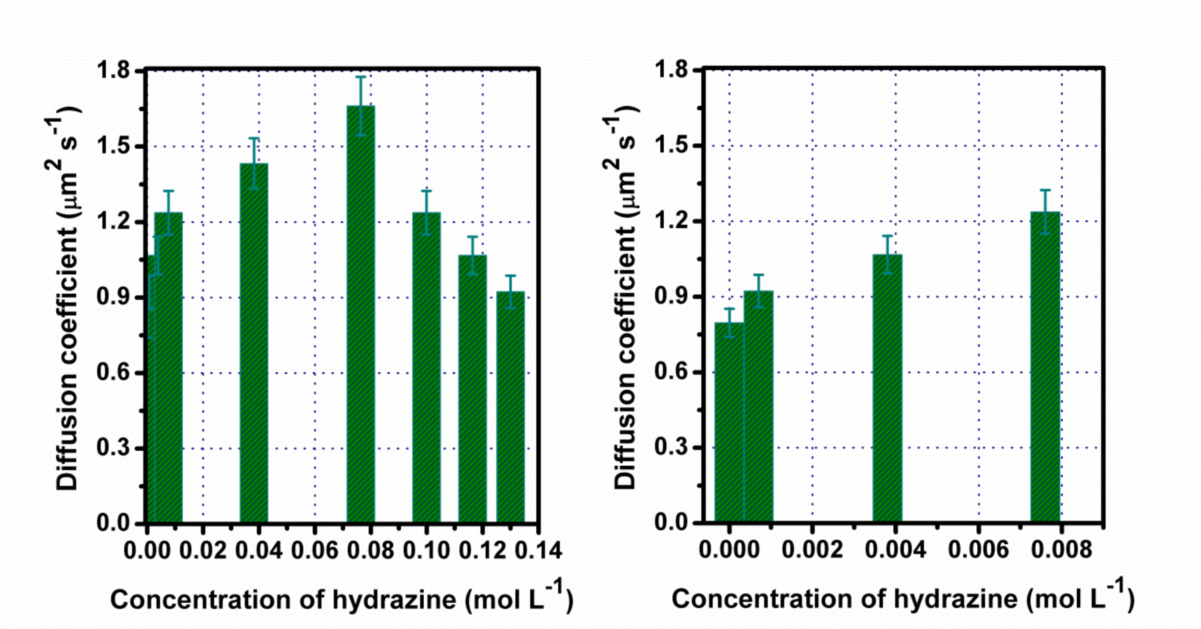

Supplementary Figure 2: Diffusion coefficient of SOMs against concentration of hydrazine. Right graph is the zoomed in part of left one for 0.0000 to 0.0764 mol L^{-1} concentration of hydrazine.

(d) Power conversion efficiency of the SOM nanomotors

The formulae used for the calculations are given (Wang et al., 2013):

Mechanical energy output = $P_{mech} = F_{drag}.v = fv^2 = \gamma v^2$

For cylinders, $\gamma = \frac{2\pi\mu L}{\ln\left(\frac{L}{R}\right)-0.72}$.

Chemical energy input = $P_{chem} = n\Delta_r G_{\gamma}$.

Efficiency, $\eta = \frac{P_{mech}}{P_{chem}}$.

F_{drag} is the drag force on the cylindrical SOM, γ is the drag coefficient, μ is the dynamic viscosity of water, L is the length of SOM, R is its radius, v is the motor speed, n is the nitrogen gas evolution rate in units of mol/(SOM·s) and $\Delta_r G_{\gamma}$ is the Gibbs free energy of the decomposition of dithionite.

Supplementary Table 2: Power conversion efficiency of the SOM nanomotors

Concentration of hydrazine sulphate (mol L^{-1})	P_{mech} (Joules)	P_{chem} (Joules)	Efficiency, η
0.0007	6.58 X 10^{-21}	2.45 X 10^{-14}	2.68 X 10^{-7}
0.0038	3.09 X 10^{-20}	1.22 X 10^{-13}	2.53 X 10^{-7}
0.0076	1.01 X 10^{-19}	2.45 X 10^{-13}	4.12 X 10^{-7}
0.0382	1.83 X 10^{-19}	1.22 X 10^{-12}	1.50 X 10^{-7}
0.0764	2.12 X 10^{-19}	2.45 X 10^{-12}	8.65 X 10^{-8}
0.0999	3.69 X 10^{-20}	4.61 X 10^{-12}	8.00 X 10^{-9}
0.1164	2.68 X 10^{-20}	5.52 X 10^{-12}	4.85 10^{-9}

(e) Calculation of surface tension:

Surface tension can be calculated using the Kelvin equation(Skinner and Sambles, 1972; Fisher and Israelachvili, 1981) as follows. The Kelvin equation may be written in the form

$$ln\frac{p}{p_0} = \frac{2\gamma V_m}{rRT}$$

where p is the actual vapour pressure, p_0 is the saturated vapour pressure, γ is the surface tension at the (SOM+hydrazine) solution/nitrogen vapour interface, V_m is the molar volume of the liquid, R is the universal gas constant, r is the radius of droplets, T is the temperature in K.

Supplementary Table 3: Calculation of surface tension

**Concentration of hydrazine (mol L^{-1})**	**Surface tension (N m^{-1}) (from Kelvin equation)**
0.0007	-3.673×10^{-9}
0.0038	-7.339×10^{-9}
0.0076	-1.129×10^{-8}
0.0382	-1.805×10^{-8}
0.0764	-2.280×10^{-8}
0.0999	1.015×10^{-8}
0.1146	2.582×10^{-8}
0.1528	6.202×10^{-8}
0.1910	1.044×10^{-7}
0.2292	1.516×10^{-7}
0.2674	2.028×10^{-7}
0.3056	2.57310^{-7}

(f) Comparison of $\{Mo_{132}\}$ and the brown powder formed after reaction from EAS and FT-IR studies

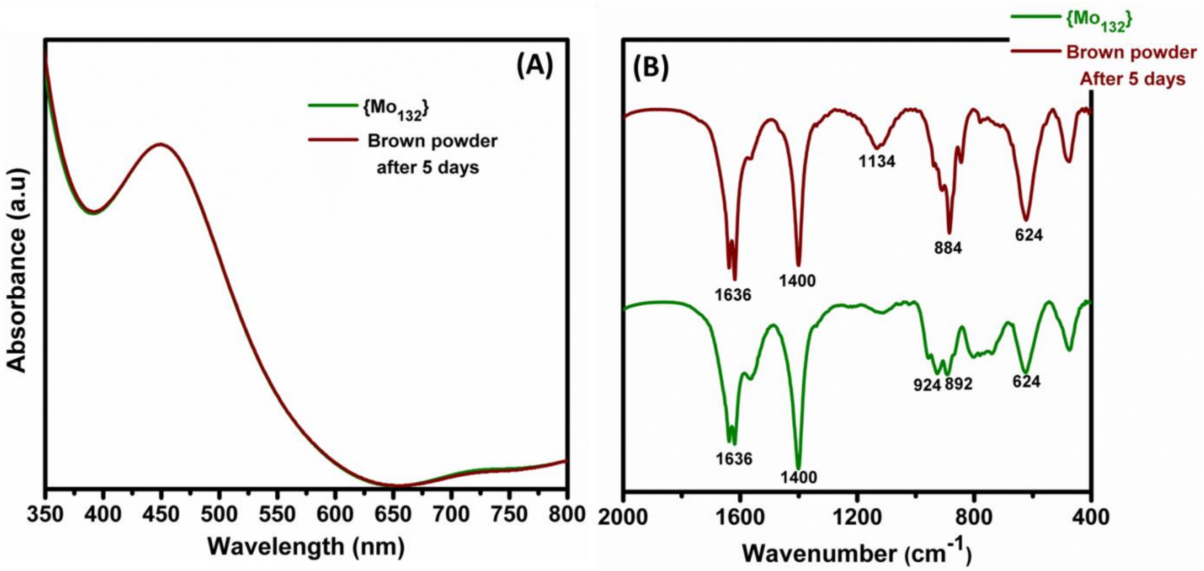

Supplementary Figure 3 (A): Electron absorption spectra and (B): FT-IR spectra of $\{Mo_{132}\}$ and the brown powder obtained after 5 days of reaction

(g) Further characterization of the brown product:

(I) Electrochemical characterization:

Cyclic voltammogram shows a cathodic peak at -0.57 V and a anodic peak at -0.4V which accounts for Mo(V)/Mo(VI).

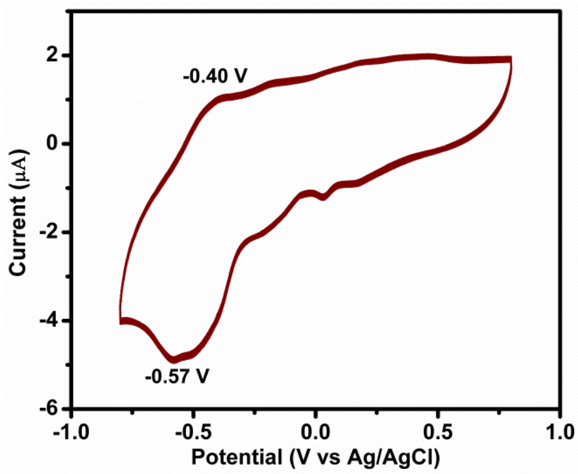

Supplementary Figure 4: Cyclic voltammogram of the brown powder obtained after reaction.

(II) Cerimetric titration of the brown powder:

To determine the number of reduced Mo centres in the brown product, we performed redox titration of the product with 0.005M Ce^{IV} solution with 0.5 M HNO_3. Ce^{IV} solution was added dropwise to a solution of 45 mg of the brown product in 50 mL water. After adding 8.4 mL of Ce^{IV} the color of the solution changes from brown to colorless. This corresponds to 2.52 X 1019 electrons. So in 45 mg of the brown product 2.52 X 1019 Mo(V) centres are present.

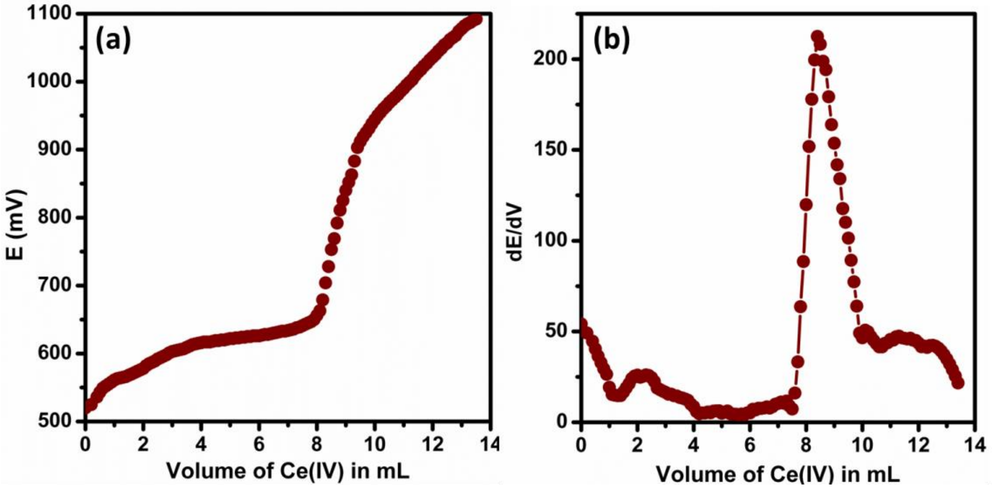

Supplementary Figure 5: Cerimetric titration of the brown product where the end point corresponds to 8.4 mL of Ce(IV) solution

(h) Formation of nitrogen gas in the reaction medium:

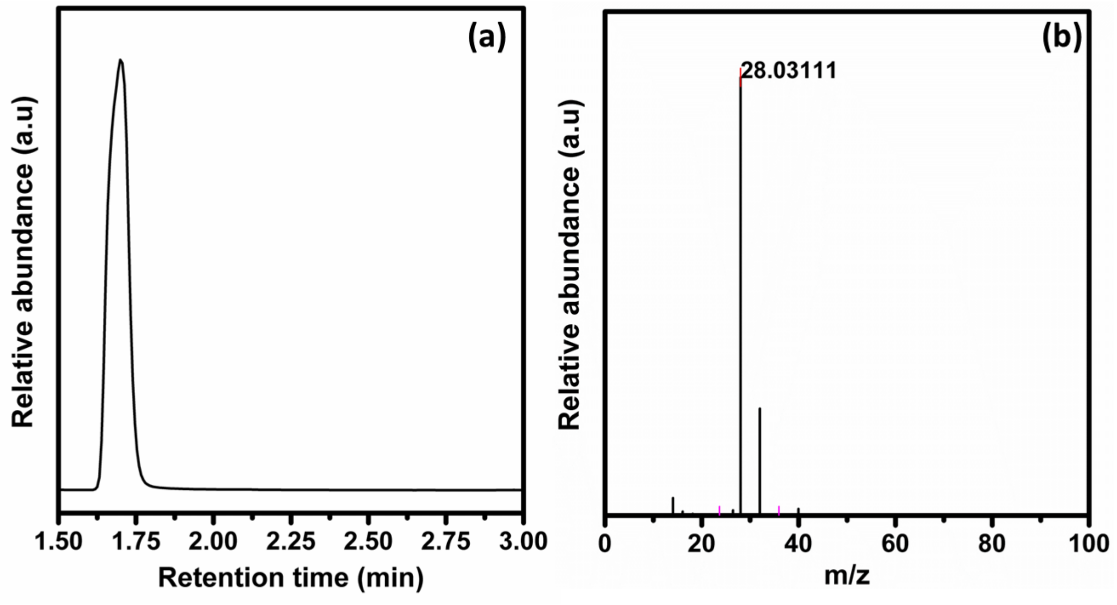

Supplementary Figure 6: GC-MS studies prove the formation of N_2 gas.

References:

Fisher, L.R., and Israelachvili, J.N. (1981). Experimental studies on the applicability of the Kelvin equation to highly curved concave menisci. *Journal of colloid and Interface Science* 80(2)**,** 528-541.

Skinner, L., and Sambles, J. (1972). The Kelvin equation—a review. *Journal of Aerosol Science* 3(3)**,** 199-210.

Wang, W., Chiang, T.-Y., Velegol, D., and Mallouk, T.E. (2013). Understanding the efficiency of autonomous nano-and microscale motors. *J. Am. Chem. Soc.* 135(28)**,** 10557-10565. doi: 10.1021/ja405135f.
